# Preoperative statins are associated with a reduced risk of postoperative delirium following vascular surgery

**DOI:** 10.1371/journal.pone.0192841

**Published:** 2018-03-23

**Authors:** Dae-Sang Lee, Mi Yeon Lee, Chi-Min Park, Dong-Ik Kim, Young-Wook Kim, Yang-Jin Park

**Affiliations:** 1 Department of Trauma Surgery, College of Medicine, The Catholic University of Korea, Seoul, Republic of Korea; 2 Department of Biostatistics, Kangbuk Samsung Hospital, Seoul, Republic of Korea; 3 Department of Critical Care Medicine, Samsung Medical Center, Sungkyunkwan University School of Medicine, Seoul, Republic of Korea; 4 Department of Surgery, Samsung Medical Center, Sungkyunkwan University School of Medicine, Seoul, Republic of Korea; University of Tampere, FINLAND

## Abstract

Delirium is a common complication of vascular surgery. The protective effect of preoperative statins on delirium after vascular surgery is controversial. The authors hypothesized that preoperative statin administration would decrease the incidence of delirium after vascular surgery. From May 2010 to May 2015, 1,132 patients underwent vascular surgery. Postoperative delirium was diagnosed from patients’ medical records. The incidence of delirium was 11.5%. The preoperative statin exposure was not associated with reduced delirium in the univariate analysis. After adjusting for covariates, preoperative statin exposure was associated with reduced delirium (OR, 0.54; 95% CI, 0.33–0.87; *p* = 0.011). This favor effect of statin for delirium was observed after propensity matching (OR, 0.59; 95% CI, 0.34–1.02; *p* = 0.060). However, the median hospital lengths of stay and in-hospital mortality were not statistically different between the two groups. CRP(C-reactive protein) levels in the unmatched population were lower in the preoperative statin group compared with the other group (p<0.001), however, there was only numerically different without statistical difference after matching (p = 0.083). Preoperative statin use was associated with a decreased incidence of postoperative delirium in patients who underwent vascular surgery. However, preoperative statin did not reduce mortality rate and hospital stay.

## Introduction

Delirium is a common complication of vascular surgery, with a frequency ranging from 19% to 42% [1–5], and is associated with a prolonged intensive care unit and hospital stay and increased morbidity and mortality. Despite several studies on postoperative delirium, the exact pathophysiologic mechanisms are not well known. Although ongoing research is examining intervention that may prevent and/or treat delirium, a favorable result on delirium is still uncertain.

Statins are compounds with pleiotropic effects, including endothelial function-enhancing, anti-inflammatory, and anticoagulant abilities [6–8]. Several studies have shown that statin exposure in the perioperative phase may reduce the incidence of delirium. However, these results are still uncertain [6–8]. Therefore, the objective of this study was to determine if preoperative statin usage was associated with a decrease in the incidence of delirium after vascular surgery. We hypothesized that preoperative statin administration would decrease the incidence of delirium after vascular surgery.

## Methods

### Study population

This was a retrospective, single-center study conducted between May 2010 and May 2015. Patients aged 18 years or older who underwent vascular surgery in the Department of Surgery, Samsung Medical Center, Sungkyunkwan University School of Medicine were enrolled. Patients were excluded from analysis based on the following criteria: the patient died within 72hrs of surgery or underwent carotid endarterectomy. Elective and emergency cases were included, such as amputation surgery, low extremity bypass, open abdominal aortic aneurysm repair, endovascular repair of aortic aneurysm (EVAR), and surgery for aortic occlusive disease. During the study period, a total of 1,608 patients underwent vascular surgical procedures. Of these, 476 were excluded. Finally, 1,132 patients were included in the study. This study was approved by the Institutional Review Board in Samsung Medical Center, and the requirement for informed consent was waived (IRB No. SMC 2015-12-169). All clinical data from patients who underwent vascular surgery were prospectively included in the vascular surgery database of our hospital, and the medical records of all patients were retrospectively reviewed. Basic demographic parameters including age, sex, comorbidities, operative details, preoperative benzodiazepine administration, smoking, alcohol, living without spouse, laboratory data, length of hospital stay, and in-hospital mortality were recorded.

### Definition and assessment of delirium

We classified patients as preoperative statin users if a statin was included in their electronic admission medication list or electronic outpatient clinic medication list. Likewise, we classified patients as preoperative beta blocker users if a beta blocker was included in their electronic admission medication list or electronic outpatient clinic medication list.

The primary outcome of this study was delirium during hospital stay. Delirium was defined as an acute deterioration of brain function characterized by a fluctuating consciousness and an inability to maintain attention, with or without accompanying agitation, and excessive motor or verbal behavior interfering with patient care, patient or staff safety, and medical therapy. Delirium was assessed by the nurse and primary physician taking part in daily patient care, on the basis of the Confusion Assessment Method (CAM). The protocol of our institution recommends the use of either haloperidol (intravenous/intramuscular administration) or quetiapine (oral administration) when delirium is assessed using the Confusion Assessment Method (S1 File). In this retrospective study, those patients who received haloperidol or quetiapine were considered to have delirium. The onset and duration of delirium were not identified in this retrospective setting.

### Statistical analysis

All data are presented as median and interquartile range (IQRs) or as the number (percentage) of patients. Data was compared using the Mann-Whitney U test for continuous variables and chi-square test or Fisher’s exact test for categorical variables. The propensity for exposure of preoperative statins was estimated without regard to outcome variables using a multiple logistic regression analysis. A nonparsimonious model that was developed including age, sex, American Society of Anesthesiologist (ASA) score over three, emergency surgery, hypertension, diabetes mellitus, Chronic obstructive pulmonary disease, Chronic renal failure, heart failure, use of preoperative benzodiazepine, use of preoperative beta blocker, living without spouse, smoking, alcohol, type of anesthesia, type of surgery, operative time over 3 hours, and anemia (hemoglobin level under 10g/dl) is detailed in Table 1. The matching algorithm used for this study was 1:1 (preoperative statin exposure and non-exposure groups). The optimal caliper width was determined by calculating 0.3 of the standard deviation of the log of the propensity score [9]. The C-statistic for the propensity score model was 0.52 (95% CI, 0.46–0.57). Standardized differences were estimated for all the baseline covariates before and after matching, and values of less than 0.1 for a given covariate indicate a relatively small imbalance (S1 Fig). This procedure yielded 291 well-matched preoperative statin exposure and non-exposure pairs. Multivariate logistic regression analysis was used to adjust for potential confounding factors in the association between preoperative statin use and postoperative delirium using the total population data set. All risk factors with an association of p<0.2 in the univariate analysis were included in the multivariate logistic model to identify risk factors for postoperative delirium. Conditional logistic regression analysis was applied to estimate the association between preoperative statin use and postoperative delirium using propensity score-matched data set. The odds ratios (ORs) and their associated 95% confidence intervals (CIs) were also calculated. For all analyses, a p value less than 0.05 was considered statistically significant. Statistical analyses were performed using STATA ver. 15 (StataCorp LP., College Station, TX, USA).

**Table 1:** Summary of baseline patient characteristics and outcomes.

	Total population			Propensity-matched population		
	Non-statin group (n = 676)	Statin group (n = 456)	p	Non-statin group (n = 291)	Statin group (n = 291)	p
Age (years)*	68 (58–74)	70 (63–75)	0.004	69 (61–75)	70 (61–74)	0.95
Male	572 (84.6)	393 (86.2)	0.47	256 (88.0)	251 (86.3)	0.54
Current smoker	188 (27.8)	97 (21.3)	0.01	69 (23.7)	68 (23.4)	0.92
History of alcohol	348 (51.5)	187 (41.0)	0.001	142 (48.8)	140 (48.1)	0.87
Living without spouse	101 (14.9)	61 (13.4)	0.46	33 (11.3)	39 (13.4)	0.45
Hypertension	421 (62.3)	368 (80.7)	<0.001	208 (71.5)	205 (70.5)	0.78
Diabetes mellitus	215 (31.8)	214 (46.9)	<0.001	121 (41.6)	110 (37.8)	0.35
COPD	48 (7.1)	44 (9.6)	0.12	27 (9.3)	27 (9.3)	>0.99
Chronic renal failure	62 (9.2)	102 (22.4)	<0.001	45 (15.5)	38 (13.1)	0.41
Heart failure	10 (1.5)	35 (7.7)	<0.001	9 (3.1)	11 (3.8)	0.65
Preoperative benzodiazepine	14 (2.1)	13 (2.9)	0.40	6 (2.1)	7 (2.4)	0.78
Preoperative beta-blocker	44 (6.5)	198 (43.4)	<0.001	43 (14.8)	38 (13.1)	0.55
General anesthesia	652 (96.4)	432 (94.7)	0.16	281 (96.6)	276 (94.9)	0.31
ASA score≥3	251 (37.1)	214(46.9)	0.001	128 (44.0)	121 (41.6)	0.56
Emergency surgery	92 (13.6)	33 (7.2)	0.001	28 (9.6)	27 (9.3)	0.89
Type of surgery			0.15			0.27
EVAR	113 (61.1)	72 (38.9)		51(51.0)	45 (49.0)	
Aortic occlusive disease	37 (68.5)	17 (31.5)		18 (54.6)	14 (45.4)	
Lower extremity bypass	270 (57.7)	198 (42.3)		107 (45.5)	129 (54.5)	
Open aortic aneurysm	167 (64.0)	94 (36.0)		71 (57.7)	53 (42.3)	
Amputation surgery	89 (54.3)	75 (45.7)		44 (48.4)	48 (51.6)	
Operative time (> 3hrs)	395 (58.4)	248 (54.4)	0.18	152 (52.2)	157 (54.0)	0.68
Anemia (Hemoglobin <10)	126 (18.6)	120 (26.3)	0.002	58 (19.9)	65 (22.3)	0.48
Outcomes						
Delirium	86 (12.7)	44 (9.6)	0.11	39 (13.4)	25 (8.6)	0.064
CRP*	11.9 (6.7–18.9)	10.3 (6.0–16.5)	0.02	11.4 (6.4–18.6)	10.3 (5.8–16.0)	0.083
hospital LOS(days)*	11 (9–15)	11 (9–17)	0.10	11 (9–18)	11 (9–15)	0.51
In-hospital mortality*, ^#^	11 (1.6)	4 (0.9)	0.28	6 (2.1)	1 (0.3)	0.123

Data are presented as n (%) or *median (IQR). CRP was maximal value within postoperative day 7. COPD, Chronic obstructive pulmonary disease; ASA, American Society of Anesthesiologist; EVAR, endovascular repair of aortic aneurysm; CRP, C-reactive protein; LOS, length of stay; IQR, interquartile range.

# fisher’s exact test

## Results

### Baseline patient characteristics: Overall population

Of the 1,132 patients, the predominant gender was male (85.2%). The median age was 69.0 years [Interquartile range (IQR), 60–74]. Emergency operation was performed in 125 patients (11.0%). The low extremity bypass was the most common type of vascular surgery, followed by open aortic aneurysm repair, EVAR, amputation surgery, and surgery for aortic occlusive disease. Baseline characteristics are shown in Table 1. The incidence of delirium was 11.5%. Delirium in the preoperative statin exposure group was less common compared with the non-exposure group, though without statistical significance (p = 0.112). However, there were significant differences in the baseline characteristics of the two groups, including age, comorbidity, smoking, alcohol, ASA score, emergency operation, anemia, and preoperative beta-blocker usage. CRP levels were lower in the preoperative statin exposure group compared with the non-exposure group (p = 0.018). CRP levels were also higher in patients experiencing delirium compared with those who did not (14.5 mg/dl vs 10.8 mg/dl, p<0.001, S1 Table).

### Propensity matched population

**Fig 1:**
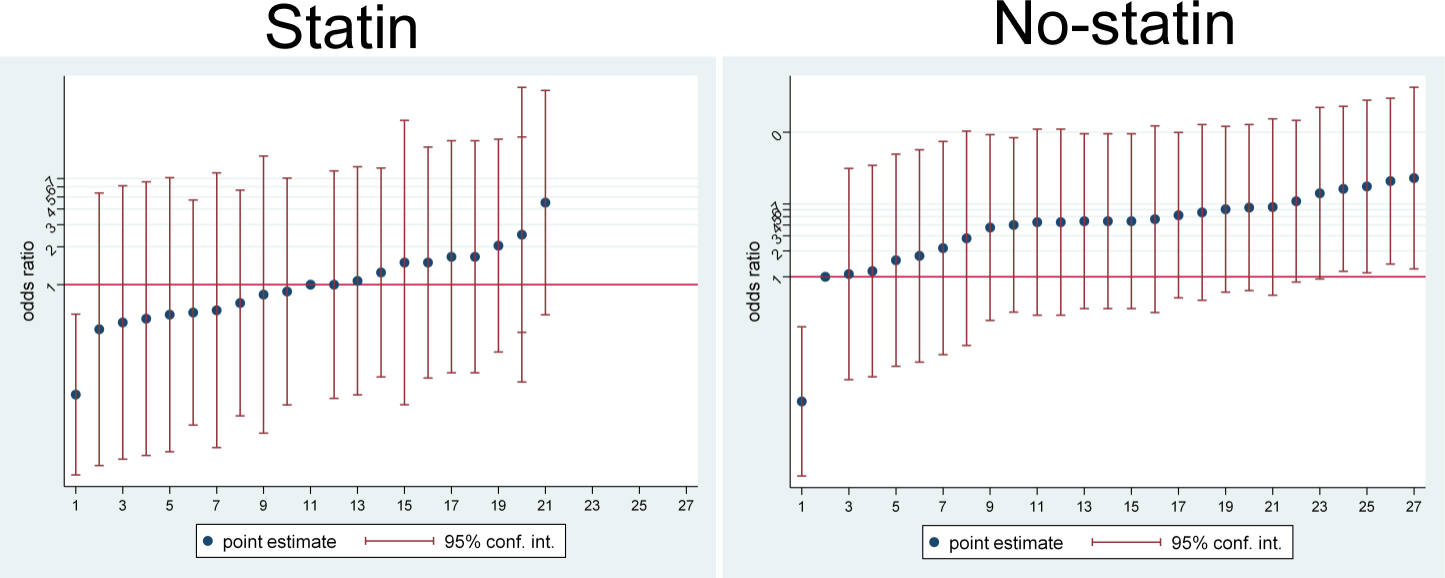
CRP level and delirium according to statin usage in the propensity matched data. The odds for delirium that was associated with preoperative statin usage is indicated by the blue circles (with corresponding 95% Cis indicated by vertical line). Result are shown according to the CRP level (x-axis).

After performing propensity-score matching for the overall population, 291 matched pairs of patients were selected (Table 1). There were no significant differences in baseline clinical, medication, or operative-related characteristics between the two groups for the propensity-matched population. Among the 582 matched subjects, the incidence of delirium was 11.0%. The preoperative statin exposure group had a lower incidence of delirium compared with the non-exposure group (8.6% vs 13.4%). The median hospital lengths of stay and in-hospital mortality were not statistically different between the two groups. CRP levels were numerically lower in the preoperative statin exposure group compared with the non-exposure group without statistical difference (p = 0.083). Also there was no statistical difference between the patients with delirium and the patients without delirium (p = 0.094, S1 Table). The effect of preoperative statin for prevent delirium was more dominant at the lower CRP interval (Fig 1).

### Multivariate analysis

Preoperative statin was not associated with delirium in the crude model (OR, 0.73; 95% CI, 0.50–1.08; p = 0.113, Table 2). This association was remain after adjusting for operation related variables such as operation time, type of surgery, emergency, type of anesthesia (adjusted OR, 0.76; 95% CI, 0.52–1.13; p = 0.181, Table 3). After adjusting for patients related variables such as hypertension, diabetes mellitus, COPD, Chronic renal failure, heart failure, ASA score and anemia, preoperative statin was statistically associated with delirium. The C statistic increased significantly when the patients related variables were incorporated in to a model with established risk factors compared to a priori model (C statistic Model 1 vs Model 2, 0.658 vs 0.723; p = 0.003). Final regression model included an additional factor with an association of p value less than 0.2 at the univariate analysis (Table 1). After adjusting for additional confounding factors, preoperative statin was associated with delirium (Table 3). The C statistic increased significantly in the final model compared to model 2 (C statistic Model 2 vs Model 3, 0.723 vs 0.748; p = 0.039) (S2 Fig). Other factors associated with delirium were age, ASA over 3, emergent operation, prolonged operation (Table 2).

**Table 2:** Logistic regression model for predicting delirium.

	Total population				Propensity-Matched population	
	Univariate		Multivariate		Univariate	
	OR (95% CI)	p-value	Adjusted OR (95% CI)	p-value	OR (95% CI)	p-value
Age (years)	1.05 (1.03–1.07)	<0.001	1.04 (1.02–1.07)	<0.001	1.08 (1.02–1.14)	0.005
Male	0.89 (0.54–1.46)	0.63			0.71 (0.23–2.25)	0.57
Current smoker	0.84 (0.54–1.29)	0.42	0.96 (0.60–1.54)	0.87	0.85 (0.38–1.89)	0.68
History of alcohol	0.83 (0.57–1.19)	0.31	0.98 (0.66–1.47)	0.94	0.73 (0.34–1.60)	0.44
Living without spouse	1.10 (0.66–1.83)	0.71			1.20 (0.37–3.93)	0.763
Hypertension	1.38 (0.90–2.10)	0.135	0.94 (0.57–1.57)	0.83	2.40 (0.85–6.81)	0.100
Diabetes mellitus	1.23 (0.85–1.78)	0.27	0.92 (0.59–1.43)	0.72	1.33 (0.56–3.16)	0.51
COPD	1.17 (0.62–2.21)	0.63	1.18 (0.59–2.36)	0.63	0.80 (0.21–2.98)	0.74
Chronic renal failure	1.75 (1.11–2.76)	0.016	1.03 (0.61–1.74)	0.92	2.60 (0.93–7.29)	0.069
Heart failure	1.71 (0.78–3.76)	0.181	1.34 (0.56–3.21)	0.51	2.00 (0.37–10.92)	0.42
Preoperative benzodiazepine	6.69 (3.06–14.64)	<0.001			N/A	
Preoperative beta-blocker	1.12 (0.72–1.73)	0.62	1.32 (0.77–2.25)	0.31	N/A	
Preoperative statin	0.73 (0.50–1.08)	0.113	0.54 (0.33–0.87)	0.011	0.59 (0.34–1.02)	0.060
General anesthesia	1.12 (0.44–2.88)	0.81	1.36 (0.48–3.87)	0.57	N/A	
ASA score≥3	2.76 (1.89–4.03)	<0.001	2.30 (1.50–3.51)	<0.001	2.20 (1.04–4.65)	0.039
Emergency surgery	2.42 (1.51–3.88)	<0.001	2.05 (1.22–3.43)	0.006	5.00 (1.10–22.82)	0.038
Type of surgery						
EVAR	1 (reference)		1 (reference)		1 (reference)	
Aortic occlusive disease	2.61 (0.94–7.21)	0.065	2.19 (0.74–6.54)	0.159	1.49 (0.17–12.84)	0.72
Lower extremity bypass	2.48 (1.24–4.96)	0.010	2.26 (1.06–4.81)	0.034	3.01 (0.75–12.09)	0.119
Open aortic aneurysm	2.53 (1.22–5.28)	0.013	1.43 (0.64–3.19)	0.39	2.80 (0.67–11.65)	0.158
Amputation surgery	2.71 (1.24–5.91)	0.012	6.10 (2.47–15.1)	<0.001	3.28 (0.72–15.01)	0.126
Operative time (> 3hrs)	1.90 (1.28–2.82)	0.001	2.36 (1.37–4.06)	0.002	1.42 (0.68–2.97)	0.36
Anemia (Hemoglobin <10)	1.95 (1.31–2.89)	0.001	1.41 (0.91–2.20)	0.125	2.50 (0.78–7.97)	0.121

N/A, not available

**Table 3:** Association between perioperative statin and delirium after potential confounding factors.

Statin	OR(95% CI)	p	C-statistic
Unadjusted Model	0.73 (0.50–1.08)	0.113	0.536 (0.493–0.580)
Adjusted Model			
Model 1	0.76 (0.52–1.13)	0.181	0.658 (0.610–0.706)
Model 2	0.59 (0.38–0.90)	0.015	0.723 (0.676–0.769)
Model 3	0.54 (0.33–0.87)	0.011	0.745 (0.704–0.792)

Adjusted Model 1: adjusted for operative time, type of surgery, emergency, general anesthesia. Adjusted Model 2: adjusted for model 1 plus hypertension, diabetes mellitus, COPD, chronic renal failure, heart failure, ASA score, anemia, Adjusted Model 3: adjusted for model 2 plus age, smoking, alcohol, preoperative beta-blocker

## Discussion

In this study, we investigated the association of preoperative statin exposure with postoperative delirium in patients who underwent vascular surgery in a single center. Preoperative statin use was associated with a lower incidence of delirium in a multivariable analysis, and this trend was remained in a propensity-matched population.

The occurrence of postoperative delirium is related to comorbidities and the operation itself. However, the pathophysiology of delirium still remains unclear and only a few causative factors are firmly established [3,5,10–13]. Because there are changes in function, synthesis, and/or availability of neurotransmitters that mediate complex cognitive and behavioral changes in delirium, statins have been recently proposed as a protective cerebral therapy due to their pleiotropic effects [6,14–16]. It has been previously shown that perioperative statin use is associated with a lower incidence of postoperative delirium [6,14–16]. The paper by Ridker et al. [17] showed that the anti-inflammatory effects of statins are related to decrease C-reactive protein (CRP) levels. This finding is based on an analysis of the CARE (Cholesterol and Recurrent Events) study showing that the use of pravastatin was associated with a decrease of cerebral and coronary ischemic events [18]. We investigated the association between preoperative statin exposure and postoperative delirium and demonstrated that preoperative statin exposure was associated with a lower incidence of postoperative delirium and lower CRP levels. In the present study, CRP levels were lower in the preoperative statin exposure group compared with the non-exposure group. CRP levels were also higher in patients experiencing delirium compared with those who did not. One important function of statins is the ability to remove oxygen-induced free radicals in a concentration-dependent manner [19]. These antioxidant effects of statin have been thought to be a possible explanation for the reported decrease in chronic cerebrovascular disease, such as Alzheimer’s disease [20–22].

Redelmeir and colleagues demonstrated no effects of preoperative statin use on postoperative delirium in 284,158 consecutive patients who were admitted for elective surgery [8]. In a study on 4,659 consecutive patients undergoing coronary artery bypass grafting, Mariscalco and colleagues did not observe any decrease in postoperative delirium with preoperative statin use [7]. Although contradictory to these previous two studies, our results are consistent with those published by Katznelson et al. [6], who examined the association between preoperative statin administration and postoperative delirium in a single-center, prospective cohort study of 582 patients undergoing vascular surgery; in their study, preoperative statin administration was independently associated with a 0.56-fold decrease in the odds of postoperative delirium. Morandi et al [23] observed that statin for critically ill patients was associated with reduced delirium, despite of a short statin usage period, especially during ICU stay. Similarly, Mather et al [24] reported the favor effect of statin for delirium in the critically ill patients. Our study also showed a favorable effect of statin, and similar risk factors for delirium, such as age, higher ASA, emergent operation, and prolonged operation, likewise other studies.[4–8,23–25]

Preoperative statin use was associated with reduced incidence of postoperative delirium in patients who underwent vascular surgery [6,26]. Although these results are hypothesis-generating, our findings support the current ACC/AHA guidelines, which recommend perioperative initiation of statin use in patients undergoing vascular surgery [27]. Patients who received a statin before their operation were characterized as having comparatively high risk factors, such as old age, hypertension, diabetes mellitus, chronic renal failure, and heart failure. Therefore, the observed effects of statins in our study may arise from differences in baseline characteristics. To deal with this, we conducted multivariate analysis and propensity score-matching to adjust the differences in baseline characteristics between groups [28]. After propensity score-matching, baseline characteristics were well balanced between two groups, and the result was similar in all patients and the propensity-matched population [28]. Moreover, patients who died within 72hrs of surgery were not included in this study by design, which reduced possible bias due to differences in baseline characteristics between the groups. The patients who died within 72hours of surgery were unstable due to operation itself and their poor preoperative condition. Because we included a broad spectrum of patients with a wide age range and did not limit the list of vascular procedures to open aortic surgery and peripheral bypass surgery, the incidence of delirium in the current study was lower compared with previously reported studies, which included only elderly patients [3] or a specific vascular surgery [2,4]. The larger sample size and real-world practices, such as administration of haloperidol to control delirium, are additional strengths of our study.

### Limitations

This study has several limitations. We used propensity score matching analysis and multivariate analysis to adjust potential confounders, but we could not modified unmeasured variables. For example, data on specific statin choice and dosing is missing. Also, we do not know how long statins were used before the operation. In current real-world practice, not all patients visit a primary care physician before operation. This means that it may be left to the surgeon to start statin therapy prior to surgery, which may not consistently occur [29]. Further, the optimal timing to start a statin is unknown. It was also unclear how much time a patient scheduled for vascular surgery could spend on a statin and still get these benefits [29]. Second, the retrospective nature of this study may have underestimated the true incidence of postoperative delirium. In this retrospective study, the patients who received haloperidol or quetiapine were considered to have delirium. This definition may have also underestimated the true number of patients who had delirium. Although, the administration of haloperidol and quetiapine was followed by the recommendation of a delirium protocol in our institution, these medications were mostly used in hyperactive delirium cases. Hypoactive delirium may be ignored in many cases, not only in our institution, but also many other hospitals. These limitations should be addressed in future trials investigating postoperative delirium.

## Conclusions

Preoperative statin use was associated with a decreased incidence of postoperative delirium in patients who underwent vascular surgery. CRP levels were lower in the preoperative statin group compared with the other group. However, preoperative statin did not reduce mortality rate and hospital stay.

## Supporting information

S1 Table Demographic data and incidence of postoperative delirium. (DOCX)

S1 Fig Distribution of covariate balance before and after matching. (TIF)

S2 Fig C statistics of each model. (TIF)

S1 File Protocol of acute management of delirium. (PDF)
