# Supplementary material for: Preoperative statins are associated with a reduced risk of postoperative delirium following vascular surgery
Source: PLoS One. 2018 Mar 23;13(3):e0192841. doi: 10.1371/journal.pone.0192841 (PMC5865715; doi:10.1371/journal.pone.0192841)
Supplement: S1 Table — (DOCX) [file pone.0192841.s001.docx]

**Supplemental Table. Demographic Data and Incidence of Postoperative Delirium**

|  | Total population | | | Propensity-Matched population | | |
| --- | --- | --- | --- | --- | --- | --- |
|  | No delirium (n=1,002) | Delirium (n=130) | p | No delirium (n=518) | Delirium (n=64) | p |
| Age (years)^*^ | 68 (59-74) | 73 (68-78) | <0.001 | 69 (60-74) | 73 (69-79) | <0.001 |
| Male | 856 (85.4) | 109 (83.8) | 0.632 | 452 (87.3) | 55 (85.9) | 0.766 |
| Current smoker | 256 (25.5) | 29 (22.3) | 0.423 | 124 (23.9) | 13 (20.3) | 0.519 |
| History of alcohol | 479 (47.8) | 56 (43.1) | 0.361 | 254 (49.0) | 28 (43.8) | 0.425 |
| Living without spouse | 142 (14.2) | 20 (15.4) | 0.710 | 65 (12.6) | 7 (10.9) | 0.712 |
| Hypertension | 691 (69.0) | 98 (75.4) | 0.134 | 363 (70.1) | 50 (78.1) | 0.181 |
| Diabetes mellitus | 374 (37.3) | 55 (42.3) | 0.271 | 201 (38.8) | 36 (46.9) | 0.213 |
| COPD | 80 (8.0) | 12 (9.2) | 0.625 | 47 (9.1) | 7 (10.9) | 0.628 |
| Chronic renal failure | 136 (13.6) | 28 (21.5) | 0.015 | 67 (12.9) | 16 (25.0) | 0.009 |
| Heart failure | 37 (3.7) | 8 (6.2) | 0.177 | 16 (3.1) | 4 (6.3) | 0.261* |
| Preoperative benzodiazepine | 15 (1.5) | 12 (9.2) | <0.001 | 6 (1.2) | 7 (10.9) | <0.001* |
| Preoperative beta-blocker | 212 (21.2) | 30 (23.1) | 0.616 | 70 (13.5) | 11 (17.2) | 0.423 |
| Preoperative statins | 412 (41.1) | 44 (33.8) | 0.112 | 266 (51.4) | 25 (39.1) | 0.064 |
| General anesthesia | 959 (95.7) | 125 (96.2) | 0.813 | 495 (95.6) | 62 (96.9) | >0.999* |
| ASA≥3 | 383 (38.2) | 82 (63.1) | <0.001 | 204 (39.4) | 45 (70.3) | <0.001 |
| Emergency surgery | 98 (9.8) | 27 (20.8) | <0.001 | 43 (8.3) | 12 (18.8) | 0.007 |
| Type of surgery |  |  | 0.012 |  |  | 0.192 |
| EVAR | 175 (94.6) | 10 (5.4) |  | 94 (94.0) | 6 (9.4) |  |
| Aortic occlusive disease | 47 (87.0) | 7 (13.0) |  | 28 (84.9) | 5 (15.2) |  |
| Low extremity bypass | 410 (87.6) | 58 (12.4) |  | 211 (89.8) | 24 (10.2) |  |
| Open aortic aneurysm | 228 (87.4) | 33 (12.6) |  | 109 (88.6) | 14 (11.4) |  |
| Amputation surgery | 142 (86.6) | 22 (13.4) |  | 76 (83.5) | 15 (16.5) |  |
| Operative time (> 3hrs) | 552 (55.1) | 91 (70.0) | 0.001 | 270 (52.1) | 39 (60.9) | 0.183 |
| Anemia (Hemoglobin <10) | 203 (20.3) | 43 (33.1) | 0.001 | 104 (20.1) | 19 (29.7) | 0.076 |
| Outcomes |  |  |  |  |  |  |
| CRP | 10.8 (6.1-17.3) | 14.5 (9.1-19.4) | <0.001 | 10.6 (6.1-16.6) | 12.6 (7.8-18.9) | 0.094 |
| hospital LOS^*^ | 11 (9-15) | 18 (11-35) | <0.001 | 11 (9-15) | 20 (11-41) | <0.001 |
| In-hospital mortality^*^ | 6 (0.6) | 9 (6.9) | <0.001 | 2 (0.4) | 5 (7.8) | <0.001 |

Data are presented as n (%) or median (IQR). *Fisher’s exact test

COPD, Chronic obstructive pulmonary disease; ASA, American Society of Anesthesiologist; EVAR, endovascular repair of aortic aneurysm; LOS, length of stay; IQR, interquartile range.
