# Supplementary figures and images for: Preoperative statins are associated with a reduced risk of postoperative delirium following vascular surgery
Source: PLoS One. 2018 Mar 23;13(3):e0192841. doi: 10.1371/journal.pone.0192841 (PMC5865715; doi:10.1371/journal.pone.0192841)

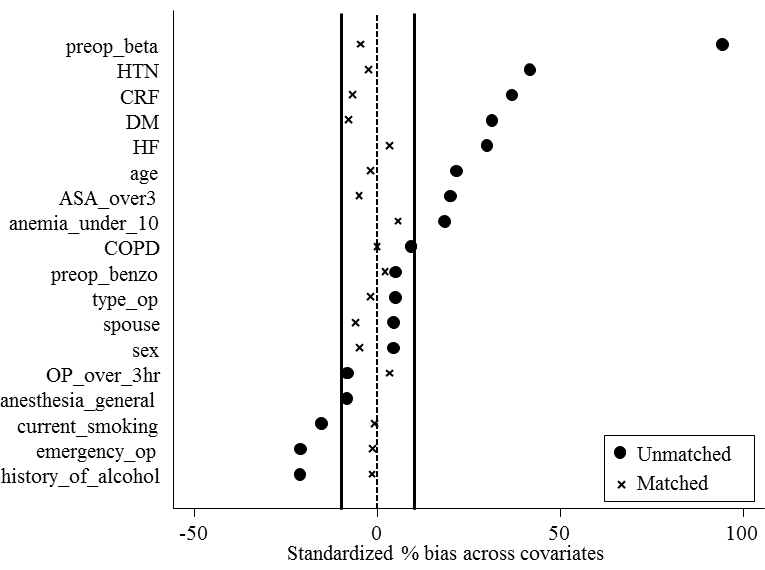

Supplement: S1 Fig — (TIF) [file pone.0192841.s002.tif]

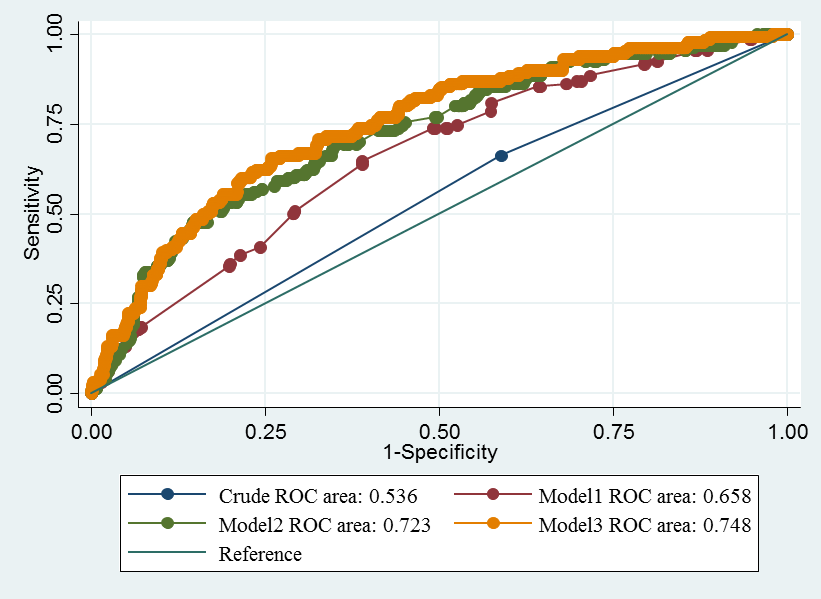

Supplement: S2 Fig — (TIF) [file pone.0192841.s003.tif]
