# Supplementary material for: Preoperative statins are associated with a reduced risk of postoperative delirium following vascular surgery
Source: PLoS One. 2018 Mar 23;13(3):e0192841. doi: 10.1371/journal.pone.0192841 (PMC5865715; doi:10.1371/journal.pone.0192841)
Supplement: S1 File — (PDF) [file pone.0192841.s004.pdf]

SMC Protocol of acute management of ICU delirium

By prof. Jeon

First proposal 2011.06.13

Algorithm for diagnosis and management of ICU delirium

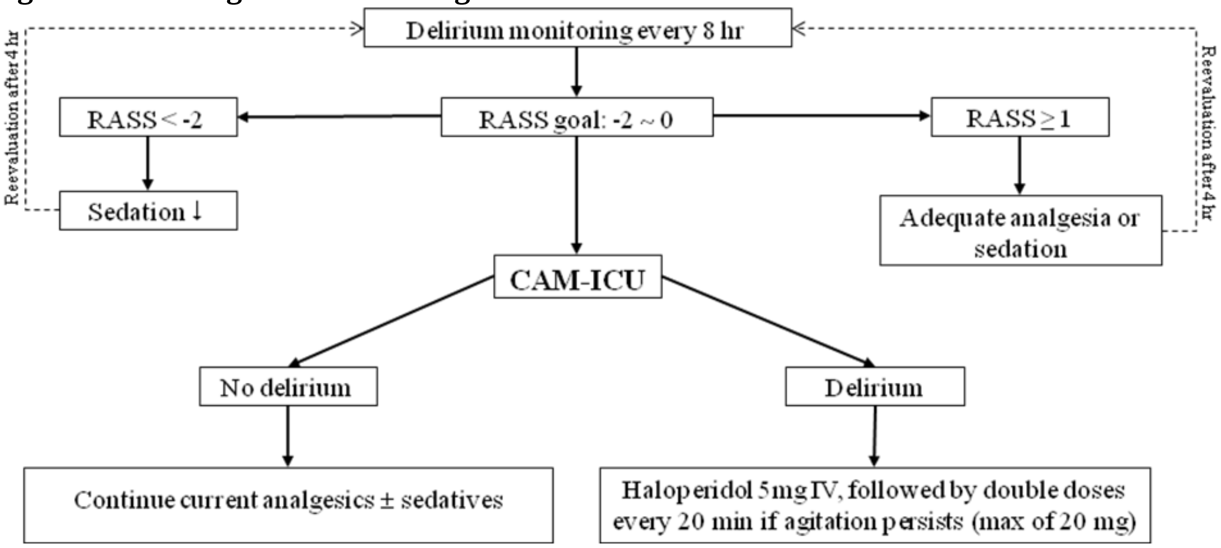

Acute management of delirium in ICU

In hyperactive delirium (agitated patients)

Haloperidol 5mg (2.5mg in the elderly of >60 yrs) intravenously, followed by double repeated doses every 20 min if agitation persists up to a maximum of 20 mg in last dose.

If response (+)

- Continuing haloperidol at 25% of the effective dose every 6 hours
- Switch to atypical antipsychotics (initiated at 50mg of quetiapine every 12 hrs)
- Titrate upward on daily basis by increments of 50mg every 12 hrs to maximum dose of 200mg every 12 hrs if patient received at lest one dose of as-needed haloperidol in the previous 24 hrs

If response (-)

- Sedation with benzodiazepin (lorazepam 1 – 4mg) IV every 10 – 20 min until acute event controlled, then q 2 – 6 hr scheduled + p.r.n.
- Reassessment of other conditions: differential diagnosis (I WATCH DEATH)

  I: infection
  W: withdrawal
  A: acute metabolic problem
  T: trauma
  C: CNS pathology
  H: hypoxia
  D: deficiencies
  E: endocrinopathies
  A: acute vascular event (stroke)
  T: toxins/drugs
  H: heavy metals

Suggested readings

1. Clinical practice guidelines for the sustained use of sedatives and analgesics in the critically ill adult. Crit Care Med. 2002 Jan;30(1):119-41.
2. Girard TD, Pandharipande PP, Ely EW. Delirium in the intensive care unit. Crit Care. 2008;12 Suppl 3:S3. Epub 2008 May 14.
3. Schiemann A, Hadzidiakos D, Spies C. Managing ICU delirium. Curr Opin Crit Care. 2011 Apr;17(2):131-40.
4. Devlin JW, Roberts RJ, Fong JJ, Skrobik Y, Riker RR, Hill NS, Robbins T, Garpestad E. Efficacy and safety of quetiapine in critically ill patients with delirium: a prospective, multicenter, randomized, double-blind, placebo-controlled pilot study. Crit Care Med. 2010 Feb;38(2):419-27.
